# Development of a recombinase-aided isothermal amplification method coupled with a lateral flow dipstick assay for the diagnosis of powdery scab in potatoes

**DOI:** 10.3389/fmicb.2025.1714852

**Published:** 2026-01-16

**Authors:** Jiahui Yang, Honghao Li, Yajie Wang, Yingqing Tang, Fengqi Lan, Yue Sun, Huanhuan Shao, Xiaojie Cheng, Xinyi He, Dongyan Liu, Yusong Jiang, Bin Yong, Xiang Tao

**Affiliations:** 1 College of Life Sciences, Sichuan Normal University, Chengdu, China; 2 Key Laboratory of Integrated Pest Management on Crops in Southwest, Ministry of Agriculture, Institute of Plant Protection, Sichuan Academy of Agricultural Sciences, Chengdu, China; 3 Research Institute for Special Plants, Chongqing University of Arts and Sciences, Chongqing, China

**Keywords:** *spongospora subterranea*, species-specificity, polymerase chain reaction, recombinase-aided amplification, molecular detection

## Abstract

Potato powdery scab is a soilborne disease caused by the fungus *Spongospora subterranea*, which belongs to the class of Plasmodiophorids and cannot be cultured. In this study, a species-specific genomic DNA fragment of *Spongospora subterranea* (2494 bp) was identified using comparative genomics methods. Polymerase chain reaction (PCR) and recombinase-aided amplification-lateral flow dipstick (RAA-LFD) base assays were then developed for the specific detection of this pathogen. Both detection methods effectively distinguished *Spongospora subterranea* from other common potato pathogens, and *Polymyxa graminis* and *Plasmodiophora brassicae*, the primary pathogens of the intercropping cruciferous and gramineous plants. The detection sensitivity of the three PCR primer pairs (SsF1/R1, SsF2/R2, and SsF3/R3) under the optimal conditions (60.5 °C; 40 cycles in a 20 μL reaction system) were 10.8 copies, 10.3 copies, and 10.6 copies, respectively. Using amplification durations of 10, 15, 20, and 25 min, the detection limits of the RAA primer and probe set (RS1F1/RI and RS1-Probe) in a 25 μL optimal reaction system were 2.51 × 10^3^, 2.51 × 10^2^, 2.51 × 10^2^, and 2.51 × 10^1^ copies, respectively. The PCR assays positively detected *Spongospora subterranea* DNA in all diseased tubers (41/41) and most samples of infested soil (27, 28, and 25 out of 31, corresponding to SsF1/R1, SsF2/R2, and SsF3/R3), whereas the RAA-LFD assay positively detected the pathogen in all tuber and soil samples when amplified at 37 °C for 20 min. The RAA-LFD outperformed PCR specifically in soil samples, mentioning performance metrics. The RAA-LFD isothermal detection assay developed herein provides a rapid, specific, and field-deployable method for diagnosing potato powdery scab in tubers and soil.

## Introduction

Potato (*Solanum tuberosum* L.) ranks as the fourth-largest food crop globally, after wheat, rice, and corn. According to the Food and Agriculture Organization of the United Nations (FAO, https://www.fao.org/home/en/) statistical data, in 2022, potato cultivation in China covered 24.35% of the world's planted area and 20.33% of the global potato production. Potato powdery scab is a soil-borne disease caused by the fungus *Spongospora subterranea*, which belongs to the order Plasmodiophorida and the family Plasmodiophoridae (Gau et al., 2013; Strydom et al., 2024). This fungus occurs in potato-growing regions across the world, and severe outbreaks can potentially cause significant yield losses (Kamal et al., 2024); even mild cases can compromise potato quality (Johnson and Cummings, 2015).

In addition to causing direct damage to potato tubers and forming root galls, *Spongospora subterranea* serves as a vector for the potato mop-top virus (Ozturk et al., 2024), making it one of the quarantine-listed pathogens under the European and Mediterranean Plant Protection Organization in the “Certification scheme for seed potatoes” (EPPO, 2023). Selecting potato cultivars with resistance to powdery scab is a key strategy for controlling this disease (Liu et al., 2023). However, as yet, no cultivar provides complete immunity to its infection yet (Yu et al., 2023). Preventing the spread of diseased seed potatoes is essential for managing diseases caused by *Spongospora subterranea*. The timely detection of *Spongospora subterranea* is crucial for effective disease management and a rapid quarantine response (DeShields et al., 2018).

Current reported diagnostic methods for potato powdery scab include the bait bioassay (Flett, 1983), enzyme-linked immunosorbent assays (ELISA) (Harrison et al., 1993; Merz et al., 2005; Wallace et al., 1995; Walsh et al., 1996), polymerase chain reaction (PCR) (Bell et al., 1999; Bulman and Marshall, 1998; Qu et al., 2006), real-time PCR (Mallik et al., 2019; Nie et al., 2021; Qu et al., 2011; van de Graaf et al., 2003; Ward et al., 2004), lateral flow immunochromatography (Bouchek-Mechiche et al., 2011), loop-mediated isothermal amplification (LAMP) (Jiang et al., 2023). However, these detection methods are either time-consuming or inconvenient for field testing. Therefore, there is an urgent necessity to develop a rapid, sensitive, cost-effective, and user-friendly molecular detection technique for soil and seed potatoes.

Recombinase polymerase amplification (RPA) is an isothermal nucleic acid amplification technique that uses recombinases derived from the T4 phage (Piepenburg et al., 2006). At room temperature, recombinases bind strongly to primer DNA, forming an enzyme-primer complex. When the primer encounters a fully complementary sequence on the template DNA, single-stranded DNA binding proteins (SSB) assist in unwinding the double-stranded template; DNA polymerase then synthesizes a new complementary strand, resulting in leading to exponential amplification of the target DNA (Lobato and O'Sullivan, 2018). RPA technology offers high sensitivity and specificity, is easy to use, requires minimal equipment, and can complete nucleic acid amplification in just 5–20 min at temperatures ranging from 30 to 42 °C. Furthermore, amplification products can be detected using lateral flow dipsticks (LFDs), thus enabling rapid and straightforward visualization. Recombinase-aided amplification (RAA) is a technology developed in China that is similar to RPA but differs only in the source of recombinase (Chen et al., 2018). The recombinase used in RAA (UvsX) is extracted from *Escherichia coli*, while the recombinase employed in RPA (T4 uvsX) is extracted from the T4 phage. Both RPA and RAA technology have been widely applied for the rapid detection of pathogenic microbes. For examples, Tang et al. developed a RAA-LFD assay for detecting potato late blight caused by *Phytophthora infestans*; the assay demonstrated a high sensitivity with a detection limit of 0.5 ag (Tang et al., 2023)., DeShields et al. developed a reverse transcription RPA (RT-RPA) test that can detect 100 of *S. subterranea* sporosori per gram of soil (DeShields et al., 2019). Ju et al. developed a RPA-LFD assay for *Neofusicoccum laricinum* (Ju et al., 2025). However, the use of RAA-LFD for the detection of *Spongospora subterranea* has not been previously documented. In this study, PCR and RAA-LFD molecular detection assays with high levels of specificity and sensitivity were developed for the diagnosis potato powdery scab. The objective of this study was to develop and validate a rapid RAA-LFD assay for *Spongospora subterranea* detection and compare its performance with PCR.

## Materials and methods

### Comparative genome analysis of *Spongospora subterranea*

The genome sequence of *Spongospora subterranea* was retrieved from the NCBI genome database (https://www.ncbi.nlm.nih.gov/datasets/genome/GCA_900404475.1/) for sequence similarity analysis against the NCBI nucleotide database (NT). First, executable BLAST+ software (v2.4.0) was downloaded from https://ftp.ncbi.nlm.nih.gov/blast/executables/blast+/LATEST/ and installed on a tower server to establish a local BLAST platform. The NT database was downloaded from https://ftp.ncbi.nlm.nih.gov/blast/db/ and then formatted by the local BLAST platform. The genomic sequences of *Spongospora subterranea* was then used as BLASTN query against the formatted NT database. The DNA fragments that did not align to any DNA sequence (excluding the sequences of *Spongospora subterranea*) were selected as candidates which were then submitted to the online BLASTN platform (https://blast.ncbi.nlm.nih.gov/Blast.cgi?PROGRAM=blastn&PAGE_TYPE=BlastSearch&LINK_LOC=blasthome) for sequence similarity analyses.

### Sequences cloning and the construction of recombinant plasmids

PCR primers, RAA primers and probes were designed by Primer Premier version 5.0 (Lalitha, 2000) based on the species-specific genomic DNA fragments, and synthesized by Beijing Tsingke Biotech Co., Ltd. Amplicons produced by the primer pairs were ligated into the pEASYB-T1 vector with a pEASYB-T1 Simple Cloning Kit (CT111-01, TransGen, Beijing, China) in accordance with the manufacturer's instructions, transformed into *Escherichia coli* BL21 chemically competent cells (CD901-02, TransGen, Beijing, China), and cultured using standard procedures. Plasmids were subsequently isolated with an EasyPure^®^ Plasmid MiniPrep Kit (EM101, TransGen, Beijing, China). Recombinant plasmids were identified by colony PCR and sequencing. Plasmid concentration was measured with a NanoDrop 400 spectrophotometer (Thermo Fisher, Wilmington, USA), and the copy number was calculated according to the concentration and molecular weight of the recombinant plasmid. A 10-fold dilution series was prepared for each plasmid.

### Establishment and optimization of a PCR reaction assay

The initial PCR cycling conditions were set according to the instructions provided with a 2 × Easy Taq PCR Super Mix (AS111, TransGen, Beijing, China): initial denaturation at 94 °C for 3 min; 30 cycles of denaturation at 94 °C for 30 s, annealing at 56 °C for 30 s, extension at 72 °C for 20 s; and final extension at 72 °C for 6 min. The PCR reaction mix contained 10 μL of 2 × Easy Taq PCR Super Mix, 1 μL of forward primer (10 μM), 1 μL of reverse primer (10 μM), 1 μL of plasmid template, and 7 μL of double distilled water (ddH_2_O). The amplicons were verified by agarose gel electrophoresis. Then, 1 μL of each diluted plasmid solution was used as a template for PCR reactions. The appropriate template concentration was selected based on the band intensity of the amplicons for subsequent optimization of PCR conditions. A gradient annealing temperature from 55 to 65 °C was created on the PCR machine, and PCR amplification was performed to identify the optimized temperature range according to band intensity. Then a 0.5 °C annealing temperature interval around the optimized temperature range was used for subsequent PCR to identify the optimal annealing temperature. Subsequently, PCR amplifications were performed using different primer concentrations (0.1, 0.2, 0.3, 0.4, and 0.5 μM) to identify the optimal concentration of primers. The optimal extension time was analyzed by amplifying with 15, 20, 25, and 30 s. A total of 25, 30, and 35 PCR cycles were performed to confirm the optimal cycle number. In all the above experiments, ddH_2_O was used as template to serve as the negative control. All reactions were performed in triplicate.

### Establishment of a RAA-LFD reaction assay

The initial amplification system was created according to the instructions of the RAA-nfo Nucleic Acid Amplification Kit (S005ZC, Zhongce, Hangzhou, China, http://hz-zc.cn/productinfo/1506906.html). During experimental operations, we added 25 μL of buffer A (10% PEG 35000), 2 μL of forward primer (2 μM), 2 μL of reverse primer (2 μM), 0.6 μL of probe (2 μM), and 10.4 μL of ddH_2_O in sequence to a tube containing reaction enzyme powder. The mixture was gently mixed and 20 μL aliquots were distributed into 0.2 mL tubes containing 2.5 μL of template DNA and 2.5 μL of buffer B [280 mM Mg(CH3COO)_2_]. The detection tube was incubated at 39 °C for 15 min and inactivated at 69 °C for 10 min. Then, 10 μL of the reaction product was submitted onto a LFD sample pad (R103ZC, Zhongce, Hangzhou, China). Subsequently, the sample pad was inserted into a 200 μL centrifuge tube containing 80 μL of ddH_2_O. Following a 5–15 min incubation period, the results were interpreted by visually inspecting the control and test lines to determine a positive outcome. When both the control and test lines were colored, the result was judged as positive. When only the control line was colored, the reaction was judged to be negative; if the test line and control line were colorless, the result was considered invalid. Subsequently, the dipstick was sealed to avoid aerosol contamination. Amplicons were analyzed by 1% agarose gel electrophoresis. ddH_2_O was used as template to serve as the negative control. Each reaction was performed in duplicate.

After diluting the recombinant plasmid to 1 × 10^10^ copies/μL, a 10-fold gradient dilution was then performed. Next, 2.5 μL of each diluted solution was used as template for RAA amplification. Amplicons were analyzed using LFD assays and 1 % agarose gel electrophoresis. The appropriate template concentration was then selected for the subsequent optimization. A total of nine RAA amplification temperatures were evaluated, ranged from 31 to 39 °C with a gradient of 1 °C. The genomic DNA of *Spongospora subterranea* was used as template according to the aforementioned RAA amplification. Amplicons were analyzed by both LFD assays and 1% agarose gel electrophoresis. Then, six RAA amplification times, including 5, 10, 15, 20, 25, and 30 min, were selected for amplification using genomic DNA as template, to determine the optimum RAA amplification time. Subsequently, amplicons were analyzed by both LFD assays and 1% agarose gel electrophoresis. In all the above experiments, ddH_2_O was used as template to serve as the negative control. Each reaction was performed in duplicate.

### Sensitivity and specificity analyses of PCR and RAA-LFD primers

The recombinant plasmids were serial diluted (10-fold) and used as a template for sensitivity analysis using the optimized PCR and RAA assay. Specifically, 1 and 2.5 μL of plasmid from each concentration were amplified by the optimized PCR and RAA reaction system, respectively. The detection limits were confirmed by 1% agarose gel electrophoresis or/and LFD assay.

The genomic DNAs of *Spongospora subterranea, Streptomyces scabies, P. infestans, P. capsici, Ralstonia solanacearum, Globodera rostochiensis, Solanum tuberosum* (potato virus-free seedlings), *Polymyxa graminis*-infested soil, and *Plasmodiophora brassicae-*infected roots were extracted using an EasyPure^®^ Genomic DNA Kits (EE101-01, TransGen, Beijing, China), an EasyPure^®^ Plant Genomic DNA Kit (EE111-11, TransGen, Beijing, China) or a TIANamp Soil DNA Kit (Tiangen Biotech, Beijing, China), according to the manufacturer's instructions. The nine genomic DNAs and a mixture of eight genomic DNAs excepting *S. subterranea* were amplified under the optimized conditions to verify the specificity of the PCR and RAA assay. The amplicons were analyzed by 1% agarose gel or/and LFD assay. In all the above experiments, ddH_2_O was used as template to serve as the negative control. Each reaction was performed in duplicate.

### Detection of diseased tubers and soil

Potato tubers with powdery scab symptoms and root-zone soil were collected from three potato-growing areas in China, including Bamei Town, Daofu County, Garze Tibetan Autonomous Prefecture, Sichuan Province (30°9'N, 101°7'E), Taian Townships, Yulong County, Yunan Province (26°8'N, 1,008'E), and Liushao Townships, Xundian County, Yunan Province (25°62'N, 102°97'E). Approximately 200 mg of lesions were scraped from diseased potato tubers. Then, genomic DNA was extracted using the EasyPure^®^ Plant Genomic DNA Kit (EE111-11, TransGen, Beijin, China). Next, soil genomic DNA was extracted using a TIANamp Soil DNA Kit (Tiangen Biotech, Beijing, China). DNA samples were used as template in an optimized PCR assay or RAA-LFD assay to detect whether the diseased tubers and soil carried *Spongospora subterranea*. Primer pairs SponF/R (Qu et al., 2011) and SsF/R (Qu et al., 2001) were used as control primers for the proven detection of *Spongospora subterranea*. DNA extracted from virus-free seedling of *Solanum tuberosum* or healthy soil sample were used as template to serve as the negative controls. All reactions were performed in triplicate.

## Results

### Identification of a species-specific genome sequence for *Spongospora subterranea*

**Figure 1:**
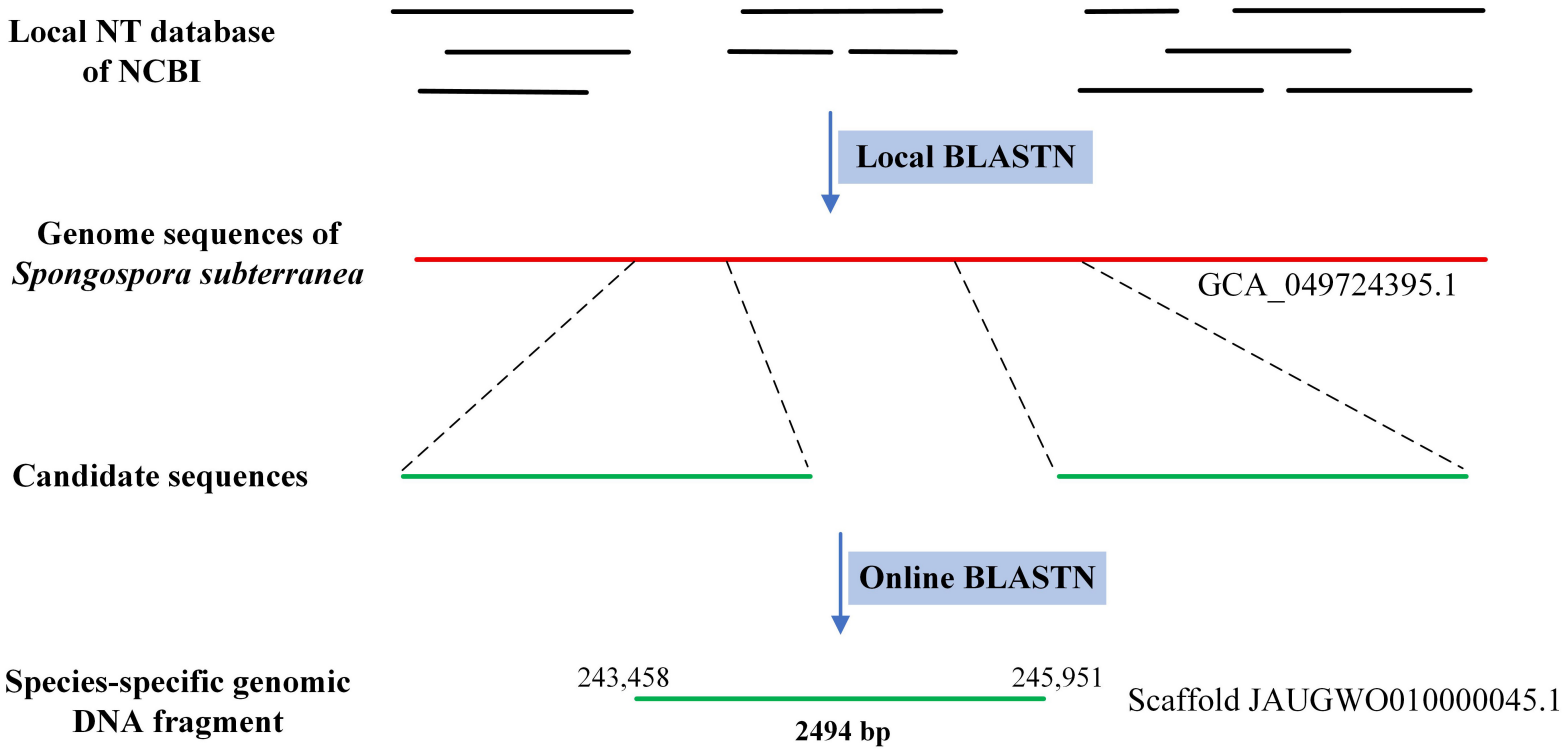
Strategy for identifying species-specific DNA fragment. The genome sequence of *Spongospora subterranea* was queried against the NT database which was formatted by the local BLAST platform. Candidate species-specific DNA sequences were then queried against the NT database by online BLASTN.

First, the genome sequence of *Spongospora subterranea* (https://www.ncbi.nlm.nih.gov/datasets/genome/GCA_900404475.1/) was queried against the NCBI nucleotide database (NT) (Figure 1). One species-specific DNA fragment (3001 bp) was identified and selected as the molecular target to develop molecular detection assays. The fragment was located in the 106,899–109,899 region on scaffold OUQQ01000006.1 and contained two small repeat regions at the 5' and 3' ends. Although several open reading frames were predicted in this fragment, the deduced proteins they encoded did not match any protein in the NCBI database. On the 16th of April 2025, a new genome was published (https://www.ncbi.nlm.nih.gov/datasets/genome/GCA_049724395.1/), which was assembled based on the sequencing reads generated by Oxford Nanopore MinION and Illumina MiSeq. The 3001 bp fragment was queried against the genome. The fragment was located in the 243,458 to 245,951 region on scaffold JAUGWO010000045.1, with a length of 2494 bp without misassembled repeat regions. No significant similarity was detected when we queried the 2494 bp fragment against the NT database.

### Construction and optimization of PCR detection assay

Three PCR primer pairs (SsF1/SsR1, SsF2/SsR2, SsF3/SsR3) were designed based on the 2494 bp fragments (Table 1). The corresponding amplicons were ligated with a linearized T-vector, resulting in three recombinant plasmids (pEASY-Ss1, pEASY-Ss2, and pEASY-Ss3). The concentrations of the three plasmids were 136.3, 66.4, and 121.1 ng/μL, corresponding to 3.2 × 10^10^, 1.5 × 10^10^, and 2.7 × 10^10^ copies/μL, respectively. The concentration of the pEASY-Ss1 were adjusted to approximately 1 × 10^10^ copies/μL, 10-fold gradient diluted and amplified by 30 cycles of PCR. The resultant amplicons were clearly observed on an agarose gel when the plasmid concentration was 10^8^, 10^7^, 10^6^, 10^5^, 10^4^, and 10^3^ copies/μL (Supplementary Figure S1). Therefore, 1 × 10^3^ copies/μL of plasmid was selected for subsequent optimization.

**Table 1:** The primers and probe used for the detection of *Spongospora subterranean*.

**Primer names**	**Tm (°C)**	**Sequence (5'to 3')**	**Amplicon length (bp)**	**References**
SsF1	61.4	CCTCGTCAATGTAAAGCCGTT	274	This study
SsR1	59.5	TCTAAATCCCCAGAATCAGTCAC		
SsF2	58.8	GATAGCGGTTTACTCAGCCC	358	This study
SsR2	59.0	TGACCTTAGCGATGATAGCACT		
SsF3	65.4	ACCTGTGTCAACCATCTCGCCT	624	This study
SsR3	58.2	GTTATCTCCATTACAATCTGCCA		
SPONF	64.4	CTTTGAGTGTCGGTTTCTATTCTCCC	138	(Qu et al., 2011)
SPONR	65.1	GCACGCCAATGGTTAGAGACG		
SsF	64.2	GTCGGTTCTACCGGCAGACC	434	(Qu et al., 2001)
SsR	65.1	GCACGCCAATGGTTAGAGACG		
Psp1	58.3	TAGACGCAGGTCATCAACCT	472	(Legrève et al., 2003)
Psp2rev	65.7	AGGGCTCTCGAAAGCGCAA		
Pb-in3-F	60.8	TACAGGAGCTGGTCCTTCCA	114	(Zhang et al., 2024)
Pb-in3-R	61.9	CGCCACACTAGCATTCAAGC		
RS1F1		TCTCCTCGTCAATGTAAAGCCGTTTGTCCT	271	This study
RS1R1		Bio-TCCCCAGAATCAGTCACCTTTCCACAGTTA		
RS1-Probe		FAM-ATCGACCTCTTTTGTTAAATGAAACATCAC-(THF)-CGGATTACGTTTGGA-(SpacerC3)		

Based on the recommended melting temperature (Tm) of the primers, eight temperatures (65.0, 64.3, 63.0, 61.1, 58.8, 56.9, 55.7, and 55.0 °C) were selected for optimization of the Tm. Analysis revealed that the three primer pairs had a wide range of suitable Tm. The optimal Tm for SsF1/SsR1 and SsF3/SsR3 was 55.0, 55.7, 56.9, 58.8, and 61.1 °C. At 65 °C, the SsF2/SsR2 amplicon exhibited a slightly lower band intensity than at other temperatures (Supplementary Figure S2). Subsequently, more precise temperature gradient experiments, with an interval of 0.5 °C, were conducted (Supplementary Figure S2). Amplifications for SsF1/SsR1 at a Tm ranging from 59.5 °C to 61.0 °C showed no notable difference in band intensity. However, from 61.5 to 63.5 °C, the band intensity gradually diminished as the Tm increased. From 63.0 to 64.0 °C, and accompanied by an increase in Tm, the band intensity produced by the SsF3/SsR3 primer pair gradually reduced. There was no obviously difference in band intensity from 60.0 to 62.5 °C. Accordingly, 60.5 °C was selected as the optimized annealing temperature for the three pairs of primers.

In addition, five different primer working concentrations, including 0.1, 0.2, 0.3, 0.4, and 0.5 μM, were used to amplify the plasmid templates (Supplementary Figure S3). The primer concentrations of 0.5, 0.4, 0.3, and 0.2 μM per primer per reaction resulted in high intensity bands. Consequently, a final primer concentration of 0.2 μM was selected. The optimal extension time was analyzed by amplification times of with 15, 20, 25, and 30 s. The amplicon produced by SsF1/SsR1 and SsF2/SsR2 showed no notable difference in band intensity at the four extension times, while the amplicon produced by SsF3/SsR3 had the highest band intensity at an extension time of 20 s. Thus, 20 s was selected as the extension time for the three pairs of primers. A total of 25, 30, and 35 PCR cycles were conducted to ascertain the optimal number of cycles. Amplification efficacy was best at 35 cycles, and satisfactory amplification outcomes were also achieved with 30 cycles of PCR. To ensure appropriate amplification, 35 cycles were selected for the following analysis.

**Figure 2:**
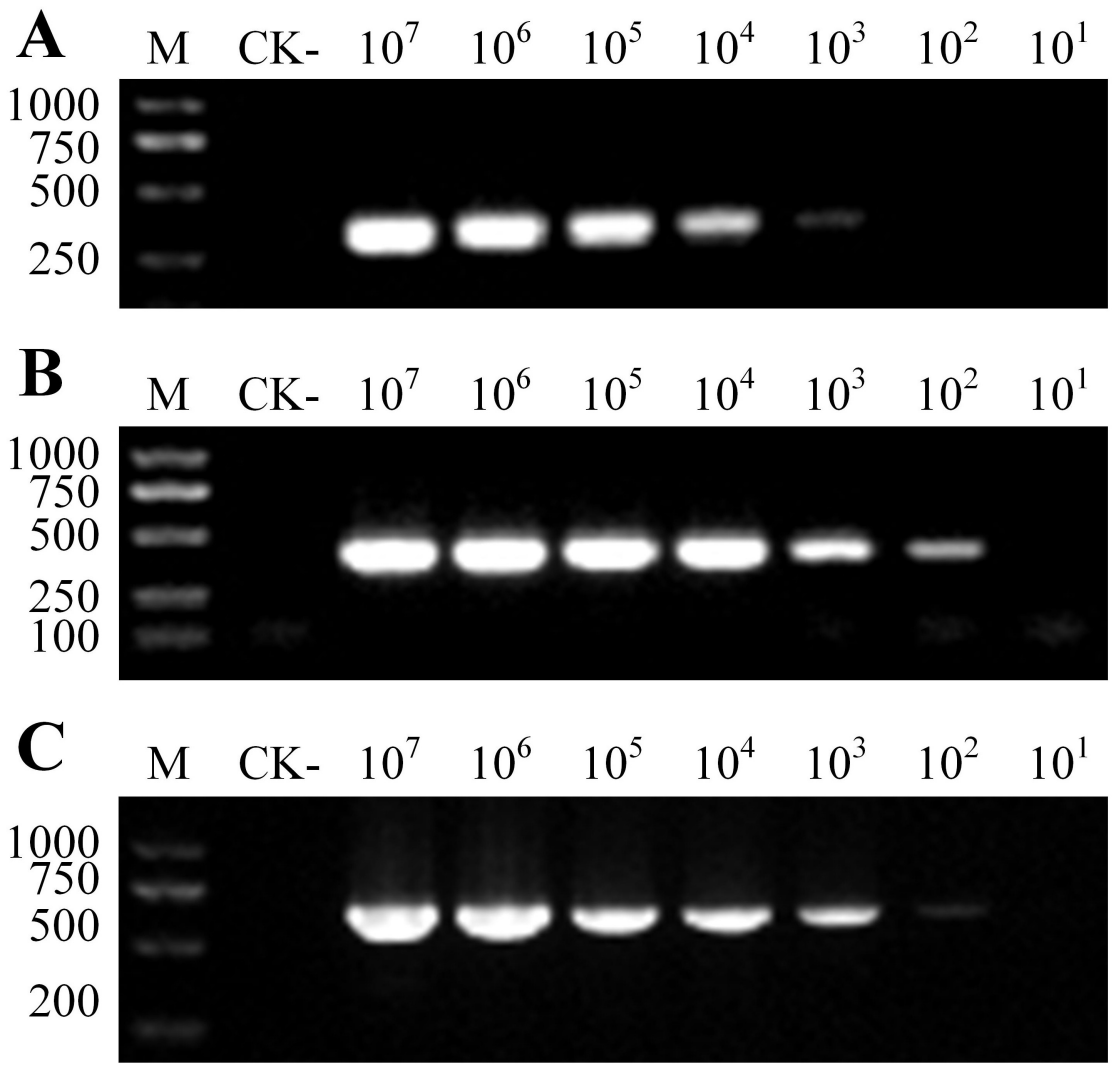
Specificity analyses of the PCR primers. **(A)** PCR amplification results for SsF1/R1. **(B)** PCR amplification results for SsF2/R2. **(C)** PCR amplification results for SsF3/R3. (1) *Spongospora subterranea*. (2) *Streptomyces scabies*. (3) *P. infestans*. (4) *P. capsici*. (5) *R. solanacearum*. (6) *G. rostochiensis*. (7) *Polymyxa graminis*. (8) *Plasmodiophora brassicae*. (9) *Solanum tuberosum*. (10) a mixture of 2–9. The PCR assay was performed in a 20 μL reaction volume and all reactions were performed in triplicate with consistent results. M: DNA marker (band size is shown in bp). CK-: negative control (ddH_2_O).

The specificity of SsF1/SsR1, SsF2/SsR2, and SsF3/SsR3 was determined using gDNA extracted from various pathogens as a template (e.g., *Streptomyces scabies, P. infestans, P. capsici, R. solanacearum, G. rostochiensis*). Except for *Spongospora subterranea* gDNA, none of the PCR reactions yielded any amplicons when using the other gDNAs as templates (Figure 2). These results indicated that all three primer pairs were highly specific. *Plasmodiophora brassicae* and *Polymyxa graminis* are closely related to *Spongospora subterranea*, and are frequently associated with potato fields, especially in intercropping systems containing cruciferous and gramineous plants. All the 52 genomes of *Plasmodiophora brassicae* were downloaded from the NCBI database (https://www.ncbi.nlm.nih.gov/datasets/genome/?taxon=37360), and formatted by the local BLAST platform (version 2.4.0). Then, the expected amplification fragments of the three primer pairs were used as BLASTN queries against the formatted database; no positive hits were generated. A total of 315 DNA sequences of *Polymyxa graminis* were downloaded from the NCBI database, and 134 RNA sequences and 497 DNA sequences of *Polymyxa graminis* were downloaded from the EMBL-EBI database. All the 945 sequences were formatted as a local database and the expected amplification fragments were queried against the database, no positive hits were generated. In addition, the gDNA of candidate samples that may have been infested by *Polymyxa graminis* and infected by *Plasmodiophora brassicae* were firstly confirmed by PCR using Psp1/Psp2rev (Legrève et al., 2003) and Pb-in3-F/Pb-in3-R (Zhang et al., 2024) as primers, and then amplified by SsF1/SsR1, SsF2/SsR2, and SsF3/SsR3 separately. It was found that the three primer pairs successfully distinguished *Spongospora subterranea* from the two other pathogens (Figure 2).

**Figure 3:**
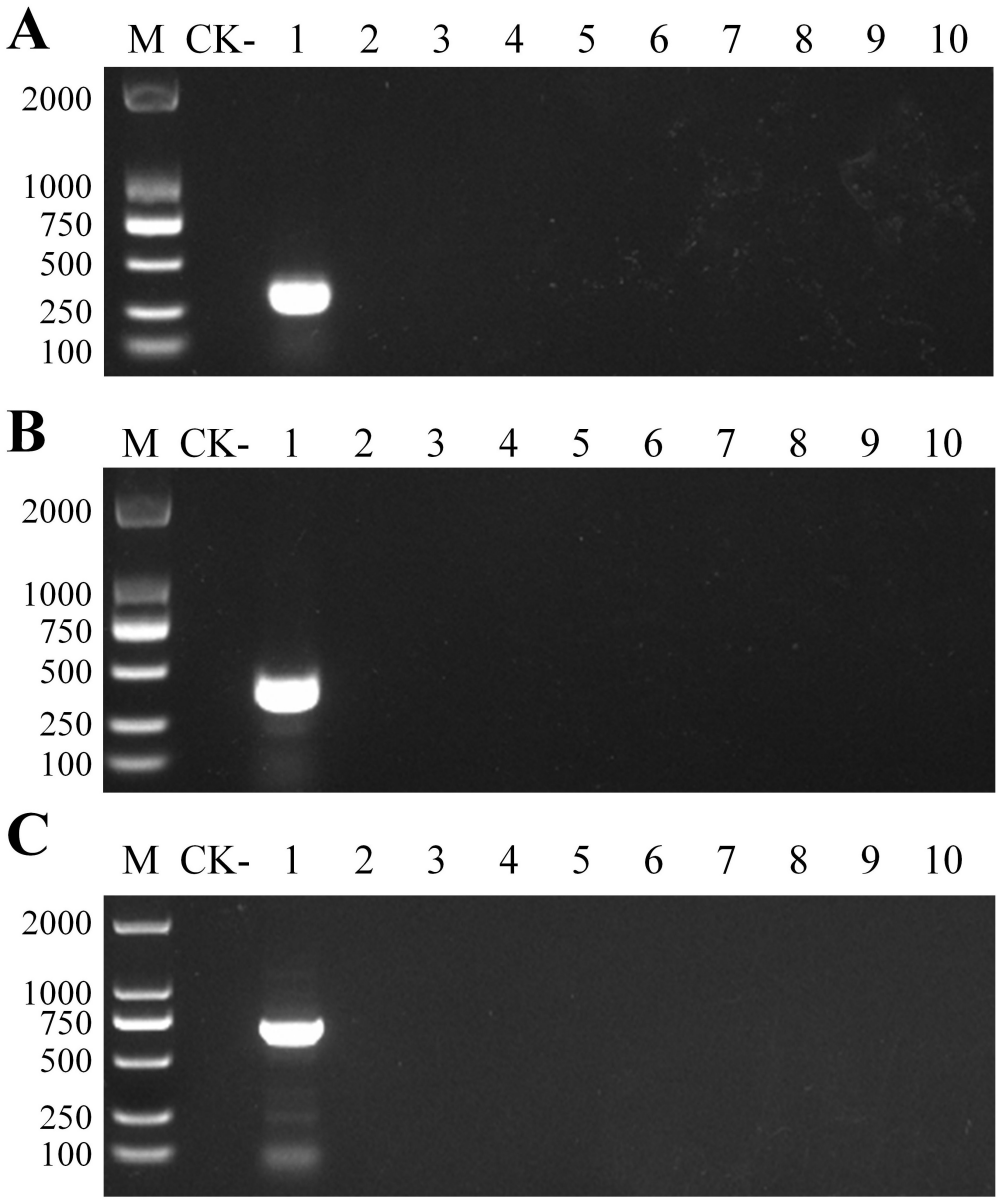
Sensitivity analysis of the PCR primers with 35 cycles. **(A)** PCR amplification results for SsF1/R1. **(B)** PCR amplification results for SsF2/R2. **(C)** PCR amplification results for SsF3/R3. The PCR assay was performed in a 20 μL reaction volume with 1 μL of template and all reactions were performed in triplicate with consistent results. M: DNA marker (band size is shown in bp). CK-: negative control (ddH_2_O).

10-fold dilutions of the recombinant plasmids (pEASY-Ss1, pEASY-Ss2, and pEASY-Ss3) were amplified using the optimized PCR assays (Figure 3). The band intensity of PCR amplicons decreased with decreasing DNA copy number. The lowest template concentrations that could be detected in a 20 μL reaction volume after 35 PCR cycles with primers SsF1/SsR1, SsF2/SsR2, and SsF3/SsR3 were 1.08 × 10^3^ copies, 1.03 × 10^2^ copies, and 1.06 × 10^2^ copies, corresponding to 4.54 fg, 0.44 fg, and 0.48 fg. When amplified upon 40 cycles of PCR, the detection limits of the three primer pairs were 10.8 copies, 10.3 copies, and 10.6 copies, respectively.

### RAA-LFD isothermal rapid detection assay

**Figure 4:**
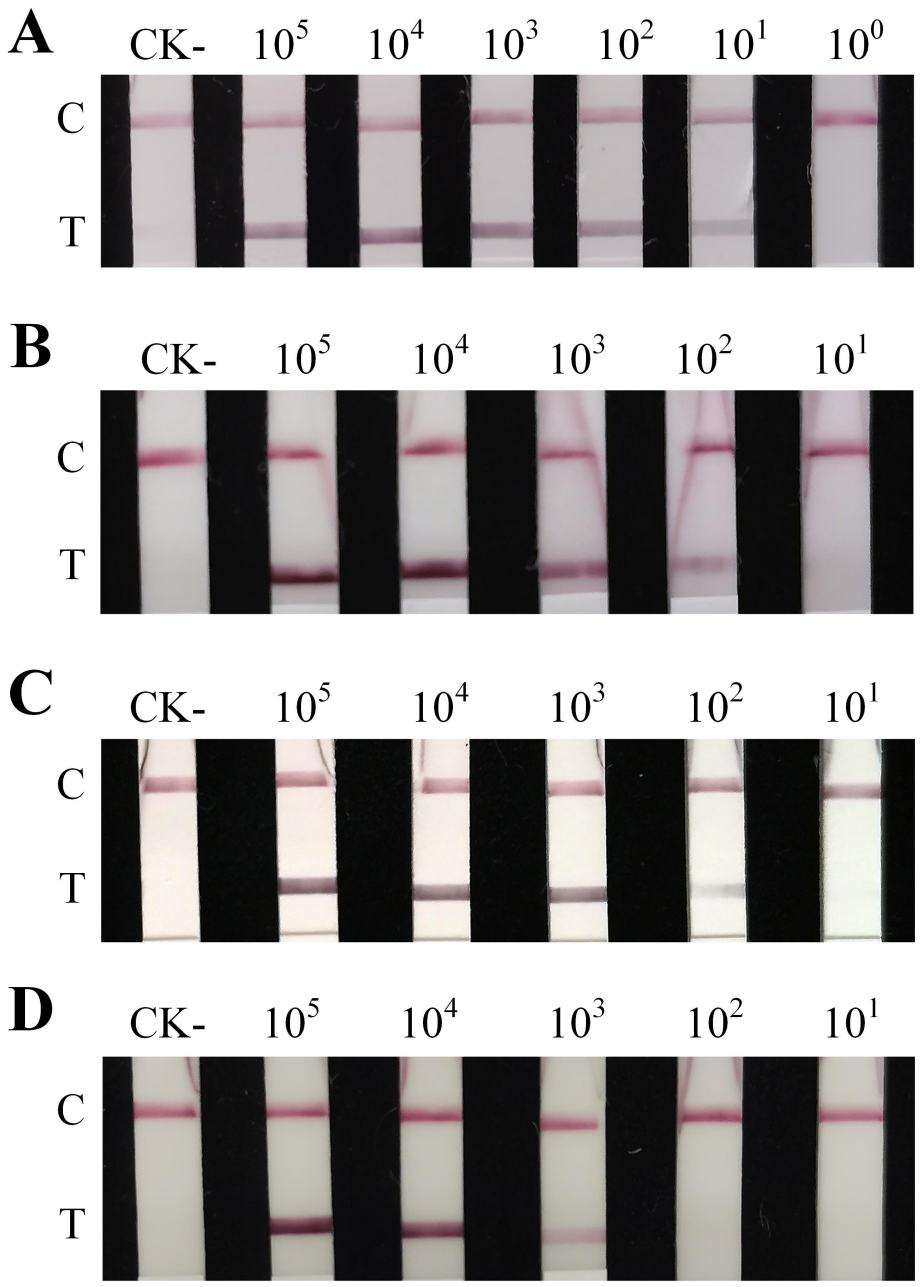
Sensitivity analysis of RAA-LFD with different amplification times. **(A)** 25 min; **(B)** 20 min; **(C)** 15 min; **(D)** 10 min. The RAA-LFD assay was performed in a 25 μL reaction volume with 2.5 μL of template. All reactions were performed in triplicate with consistent results. CK-: negative control (ddH_2_O). C: control line; T: test line.

RAA primers and probes were designed based on the identified species-specific DNA fragments. The recombinant plasmid pEASY-Ss1 was serial diluted 10-fold and used as template for RAA (Supplementary Figure S4). Stable amplicons were observed when the template concentration was 10^4^ copies/μL. Therefore, a template concentration of 10^4^ copies was selected for subsequent optimization. RAA amplification was performed at temperatures ranging from 31.0 to 39.0 °C, with an interval of 1.0 °C (Supplementary Figure S5). For all nine temperatures, the intensity of the detection bands on the LFD did not show obviously differences. However, the bands gradually weakened as the temperature decreased, as detected by agarose gel electrophoresis. For convenience, 37 °C, which is closest to axillary temperature in humans, was selected as the optimal temperature. Subsequently, pEASY-Ss1 was amplified at 37 °C with different times ranging from 5 min to 30 min, with an interval of 5 min. The longer the amplification time, the clearer the detection bands. When we extended the RAA amplification time to 10, 15, 20 and 25 min, the detection limits were 2.51 × 10^3^, 2.51 × 10^2^, 2.51 × 10^2^, and 2.51 × 10^1^ copies (Figure 4), corresponding to 10.57, 1.06, 1.06, and 0.11 fg, respectively. Amplification was performed using the gDNA from several phytopathogens and the virus-free potato seedlings as templates, at 37 °C for 20 min (Supplementary Figure S6); gDNA of *Spongospora subterranea* was successfully detected as expected, but no amplicons were observed for the other DNA templates, either on the gel electrophoresis or on LFD.

### Detection of infected tubers and soil samples by PCR and RAA-LFD assays

**Figure 5:**
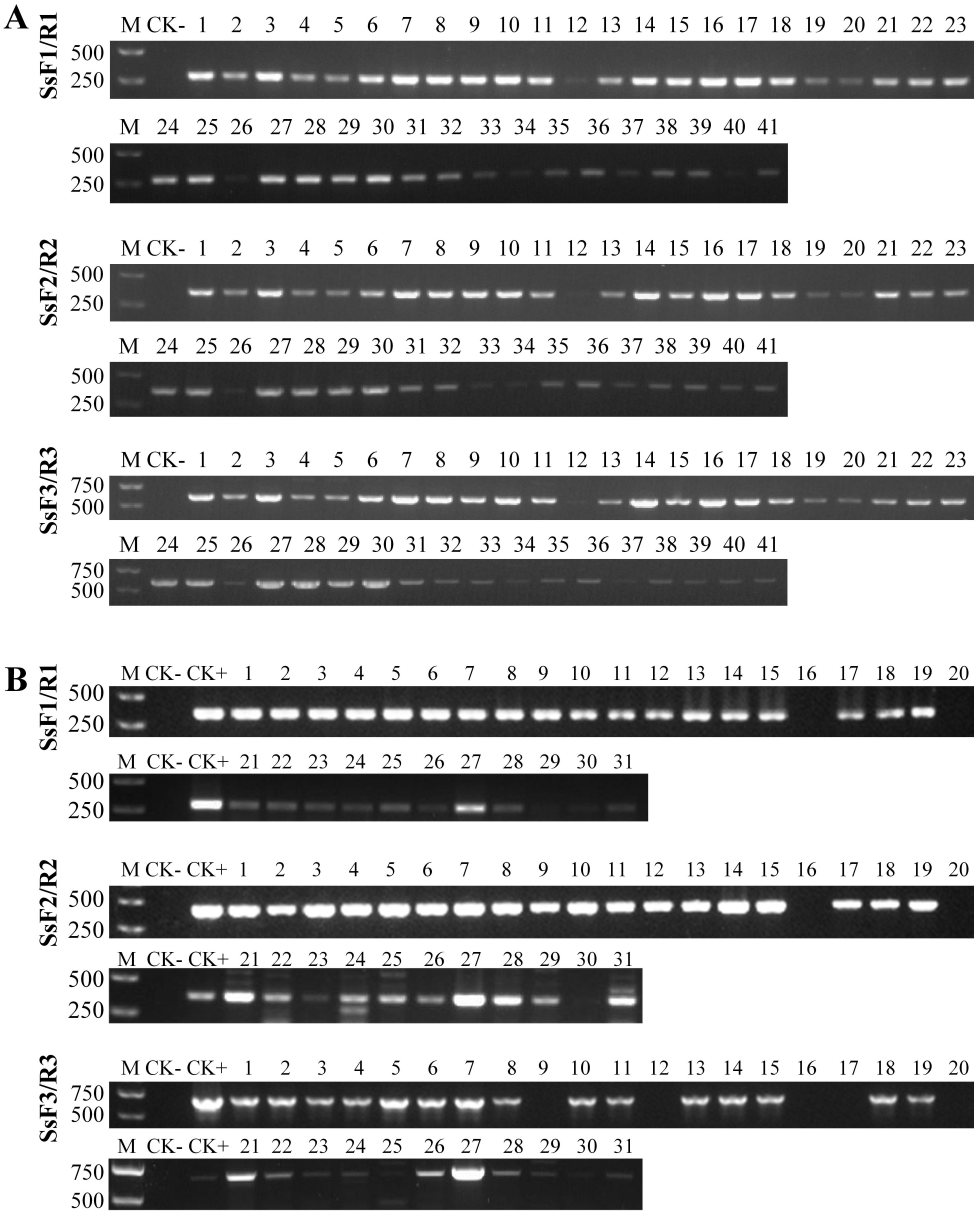
Detection of diseased potato tubers and soil samples with PCR assays. **(A)** Detection of diseased potato tubers with 30 PCR amplification cycles. **(B)** Detection of infested soil with 40 PCR amplification cycles. M: DNA marker (band size is shown in bp). CK+: positive control (*Spongospora subterranea*); CK- in subfigure A: negative control (virus-free seedling of *Solanum tuberosum*); CK- in subfigure B: negative control (healthy soil sample).

**Figure 6:**
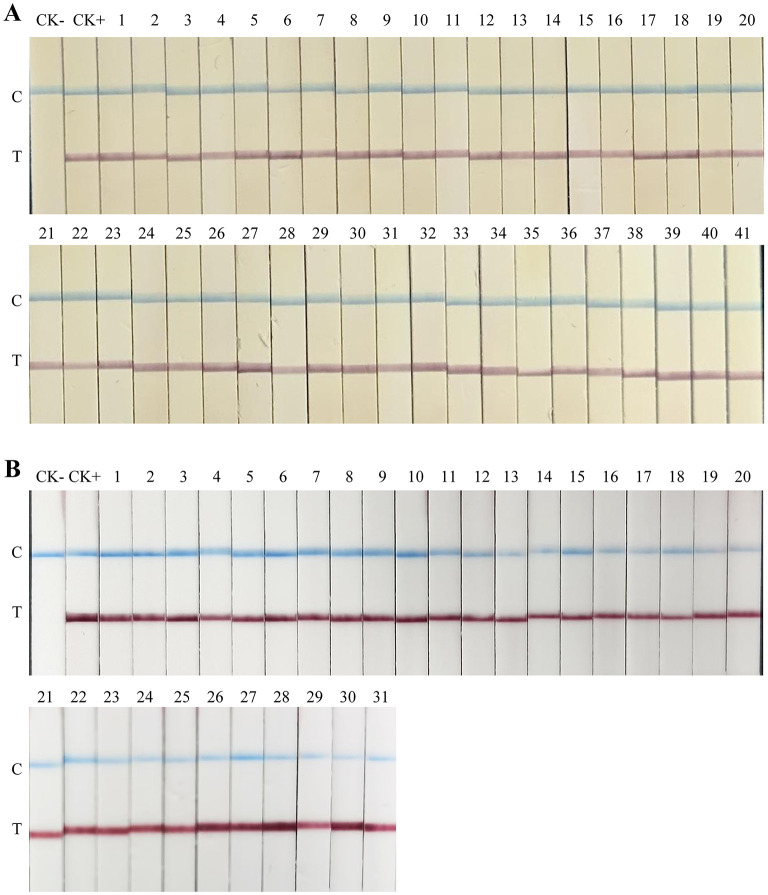
Detection of diseased potato tubers and soil samples with RAA-LFD assays. **(A)** detection of diseased potato tubers. **(B)** detection of infested soil. A was amplified for 15 min, B was amplified for 20 min. The RAA-LFD assay was performed in a 25 μL reaction volume with 2.5 μL of template. CK+: positive control (*Spongospora subterranea*); CK- in subfigure **(A)** negative control (virus-free seedling of *Solanum tuberosum*); CK- in subfigure **(B)** negative control (healthy soil sample). C: control line; T: test line. The variations in color of the test and control lines among subfigure **(A** and **B)** are due to the use of LFD from different production batches.

A total of 41 potato tubers infected with powdery scab were sampled (30°9'47”N, 101°7'23”E); gDNAs were extracted from the diseased lesions and tested using SsF1/SsR1, SsF2/SsR2, and SsF3/SsR3 primer pairs (Figure 5). The positive detection rate for the three primer pairs was 100% after 30 and 35 PCR cycles. SPONF/R (Qu et al., 2011) and SsF/R (Qu et al., 2001) were used as control primers and also yielded a 100% positive rate. Furthermore, RS1F1/R1 and RS1-Probe were employed to test the 41 field samples (Figure 6A), achieving a 100% positive detection rate after 15 min of amplification.

Next, gDNAs extracted from 31 soil samples collected from potato powdery scab-infested fields were used as templates for PCR assays and RAA-LFD assay. After 40 PCR amplification cycles, 27, 28, and 25 DNA samples tested positive using SsF1/SsR1, SsF2/SsR2, and SsF3/SsR3 as primers, corresponding to positive detection rates of 87.1%, 90.3%, and 80.6%, respectively (Figure 5). The two control primer pairs successfully detected 19 positive samples. When the soil gDNAs were amplified using the RAA assay at 37 °C for 15 min, 29 samples were identified as positive. Extending the amplification time to 20 min increased the positive detection rate to 100% (Figure 6B).

## Discussions

### Specificity of the detection assays

*Spongospora subterranea*, which belongs to the Plasmodiophorida order and the Plasmodiophoridae family (Gau et al., 2013; Strydom et al., 2024), is a soil-borne pathogen that exerts a marked effect on the global potato industry. Over recent years, several articles have reported the molecular detection methods for this pathogen. Bulman and Marshall (1998) designed PCR primers based on the ITS sequence and successfully detected *Spongospora subterranea* using PCR. Bulman and Marshall's primer, Spo1 (ATTGTCTGTTGAAGGGTG), exhibited 100% and 94% identity with the genomes of *Erwinia tracheiphila* and *Brassica rapa*, whereas Spo2 (GGTTAGAGACGAATCAGAA) exhibited 100% identity with the genomes of *Apis dorsata, A. laboriosa*, and et al. In addition, Bulman and Marshall's Spo8 and Spo9 primers also exhibited high identity with the genome of related species. In another study, DeShields et al. (2019) designed primers based on the ITS1 of *Spongospora subterranea* and developed RPA assays to detect the sporosori of *Spongospora subterranea*. However, their reverse primer exhibited 100% identity with the genomes of *Polymyxa graminis, Hillenburgia nasturtii*, et al., while their probe exhibited high identity with *Polymyxa graminis* and several other related species. The primers and/or probe used by Hernandez Maldonado et al. (2013) and van de Graaf et al. (2003) were associated with the same limitations. The Spo10/Spo11 primer pair and the SpoPro1 probe have been widely used for diagnostics and are considered to be reliable for the detection of *Spongospora subterranea* (Hernandez Maldonado et al., 2013). However, the Spo10 primer exhibits 100% identity with the DNA sequence of several fungi that cannot be cultured, and 94% identity with the DNA sequences of *Aspergillus wentii* DTO 134E9, *Sphagnum jensenii*, and *S. palustre*. Spo11 exhibits the same limitations. In certain circumstances, cruciferous plants and gramineous plants are inter-cropped with potatoes (Messiha et al., 2019). In these situations, primers designed based on the 18S rRNA gene or ITS sequence may lead to false positives. Therefore, it is important to investigate species-specific detection targets for the prevention and control of powdery scab. In this study, the genome of *Spongospora subterranea* was first submitted for multiple sequence alignment with the NCBI NT nucleotide database. We successfully identified a 2494 bp species-specific DNA fragments. Prospectively, we designed primers based on this fragment, which could avoid the false positives caused by DNA of plants, fungi, and bacteria in the soil.

The obligate biotrophic nature of the class of Plasmodiophorids pathogens makes renders them incapable of culture; this property has seriously impeded the research targeting these pathogens (Neuhauser et al., 2010). Very few closely related species of *Spongospora subterranea* have been identified. In the present study, the specificity of the detection target fragment was preliminarily verified by using the gDNA from virus-free potato seedlings and seven common pathogens as PCR templates; the PCR detection system was proven to be highly economical (Table 2). Next, the primers and probe were aligned to the genome sequences of the related species belonging to the Plasmodiophoridae. Only three species of the Plasmodiophoridae family, including *Plasmodiophora brassicae, Polymyxa betae*, and *Spongospora subterranea* (https://www.ncbi.nlm.nih.gov/datasets/genome/?taxon=37358), have reported their genomes. Sequence similarity alignments revealed that primers RS1F1 and RS1R1, along with the RS1-Probe, aligned with several loci on the genome of *Polymyxa betae*. However, at least two bases at the3' end of the primers did not match in any of the potential binding sites. The positivity of the probe was only 48.89% and 28.89% when tested with the other two genomes, respectively. Therefore, the primers and probe used in this study exhibit better species-specificity comparatively.

**Table 2:** Comparisons of different detection assays.

**Primers**	**Method**	**Time expense (h)**	**Cost per reaction (USD)**	**Sensitivity**	**References**
SsF1/R1	PCR	~1.7	~3	10.8 copies	This study
SsF2/R2	PCR	~1.7	~3	10.3 copies	This study
SsF3/R3	PCR	~1.7	~3	10.6 copies	This study
SPONF/R	PCR	>24.0	~3	100 fg	(Qu et al., 2011)
SsF/R	PCR	>24.0	~2	NA	(Qu et al., 2001)
F3/B3	PCR	~2.5	~8	60 copies	(Jiang et al., 2023)
SPO10/SPO11	qPCR	~3.0	~6	10 copies	(Hernandez Maldonado et al., 2013; DeShields et al., 2018)
SsTQ-F/R	qPCR	~3.0	~5	10 copies	(DeShields et al., 2018)
RS1F1/R1	RAA	~1.5	~7	25 copies	This study
SpITS1-2F/2R	RPA	~3.0	~6	100 sporosori/g soil	(DeShields et al., 2019)
F3/B3/FIP(F1c+F2)/BIP(B1c+B2)	LAMP	~2.0	~10	6 copies	(Jiang et al., 2023)

Time expense and cost per reaction refer to the time and cost required for all procedures following sample collection, including preprocessing, DNA extraction, and testing.

PCR, polymerase chain reaction; qPCR, quantitative polymerase chain reaction; RAA, recombinase-aided amplification; RPA, recombinase polymerase amplification; LAMP, loop-mediated isothermal amplification; USD, United States dollar.

### Sensitivity of the detection assays

Flett used tomato seedlings as baits to detect *Spongospora subterranea* in soil samples (Flett, 1983). This technique detected 10 spore balls per 150 mL of test solution. Nevertheless, this technique involved an incubation period of up to 21 days and necessitates the utilization of a microscope for the detection of sporangia, thus requiring the operator to possess extensive taxonomic expertise. Harrison et al. (1993) established an ELISA system that used polyclonal antisera against powdery scab cystosori, which was able to detect asymptomatic contamination in tubers; however, this system performed poorly for detection of spore balls in soil. Wallace et al. (1995) established a monoclonal antibody-based ELISA detection method but could only detect the presence of cystosori when unrealistically large numbers were present in the test system. Bulman and Marshall (1998) designed PCR primers based on the ITS sequence of *Spongospora subterranea*; the primer pair Spo8/9 could easy to detect 20 sporosori with approximately 2 cm^2^ of the common scab tissue.

Although PCR is fast and cost-effective, its exhibits low sensitivity, thus allowing for only qualitative detection. Furthermore, PCR cannot be conducted outside of a laboratory setting. LAMP, RAA/RPA, and other isothermal amplification methods incur shorter detection times, do not require a PCR amplifier, and provide visual results, making them more suitable for field testing and high-throughput detection. The reported two genomes of *Spongospora subterranea* (https://www.ncbi.nlm.nih.gov/datasets/genome/?taxon=70186) contains 346 and 2,340 scaffolds; however, several gaps remain in the genome sequence. Therefore, it is impossible to accurately calculate the molecular weight and copy number based on the concentration of gDNA solution. In this study, a recombinant plasmid was constructed and used as template to develop molecular detection assays. Using plasmids as templates not only avoid the problem of isolating and purifying the bacteria to be detected but also facilitates the analysis of the sensitivity. The lowest concentration of template that could be detected in a 20 μL reaction volume after 40 PCR cycles with our PCR primer pairs was ~10 copies. However, these systems relied on expensive PCR amplifier. Amplifying a template with RAA at 37 °C for 25 min, we were able to detect 25 copies in a 25 μL reaction volume; this was considerably lower than the results of the PCR and RPA assays developed by Jiang et al. (2023), Qu et al. (2011), and DeShields et al. (2019) (Table 2). However, the cost of RAA assay is still high comparatively. The future directions include: (1) developing multiplex RAA detection assays for a variety of diseases; (2) developing lower-cost visualization methods, such as the SYBR green-based method; and (3) designing portable isothermal amplification devices.

## Data Availability

The original contributions presented in the study are included in the article/Supplementary material, further inquiries can be directed to the corresponding authors.
