# Supplementary figures and images for: Development of a recombinase-aided isothermal amplification method coupled with a lateral flow dipstick assay for the diagnosis of powdery scab in potatoes
Source: Front Microbiol. 2026 Jan 16;16:1714852. doi: 10.3389/fmicb.2025.1714852 (PMC12855457; doi:10.3389/fmicb.2025.1714852)

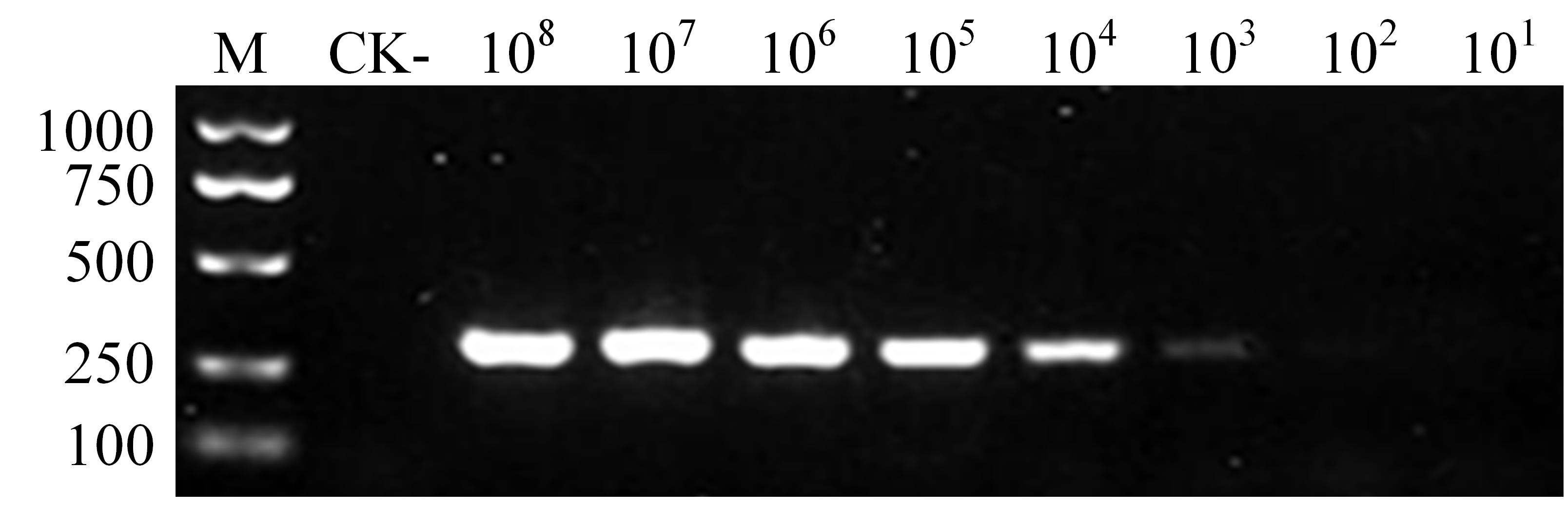

Supplement: Supplementary file 1 [file Image_1.jpeg]

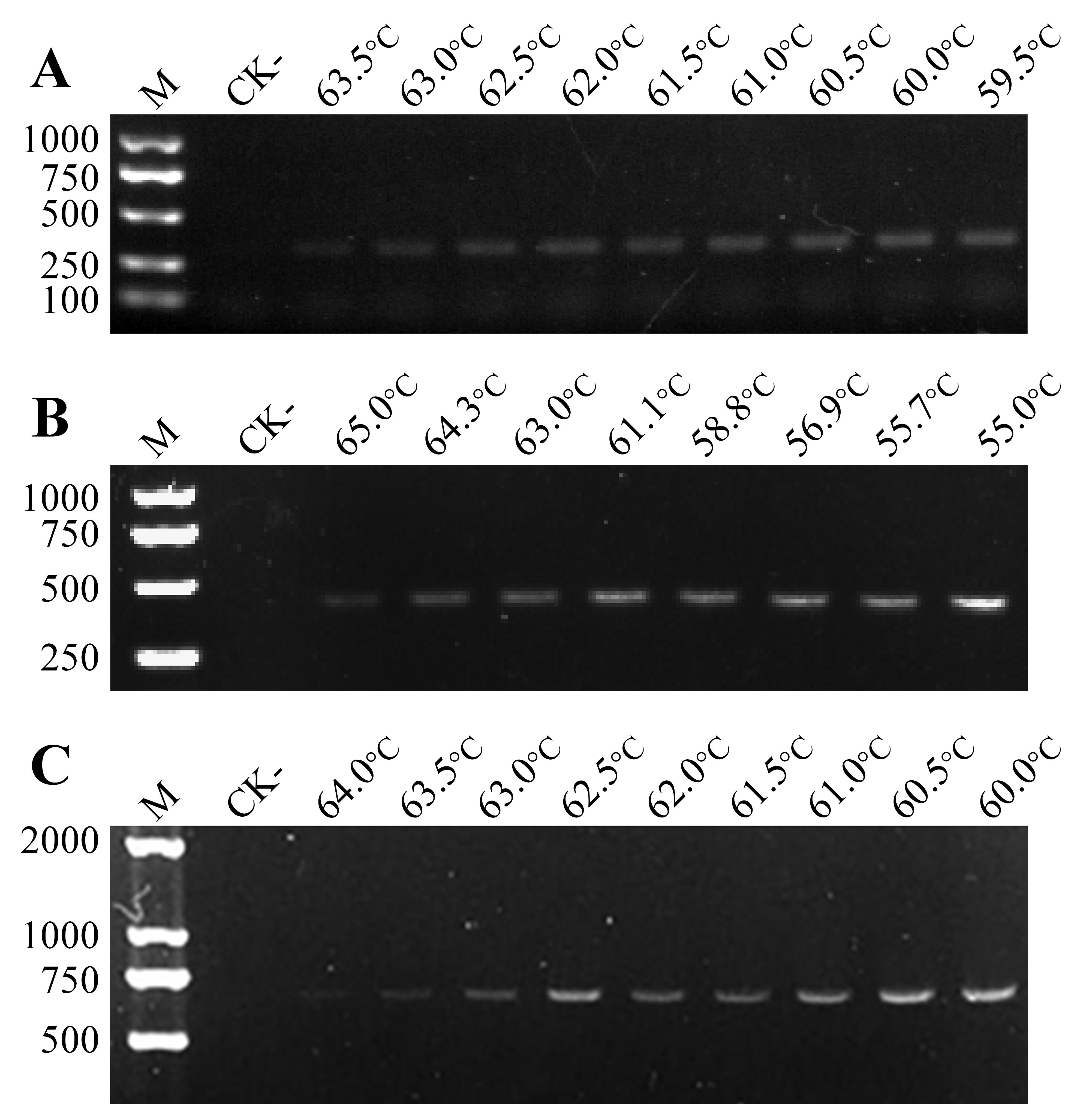

Supplement: Supplementary file 2 [file Image_2.jpeg]

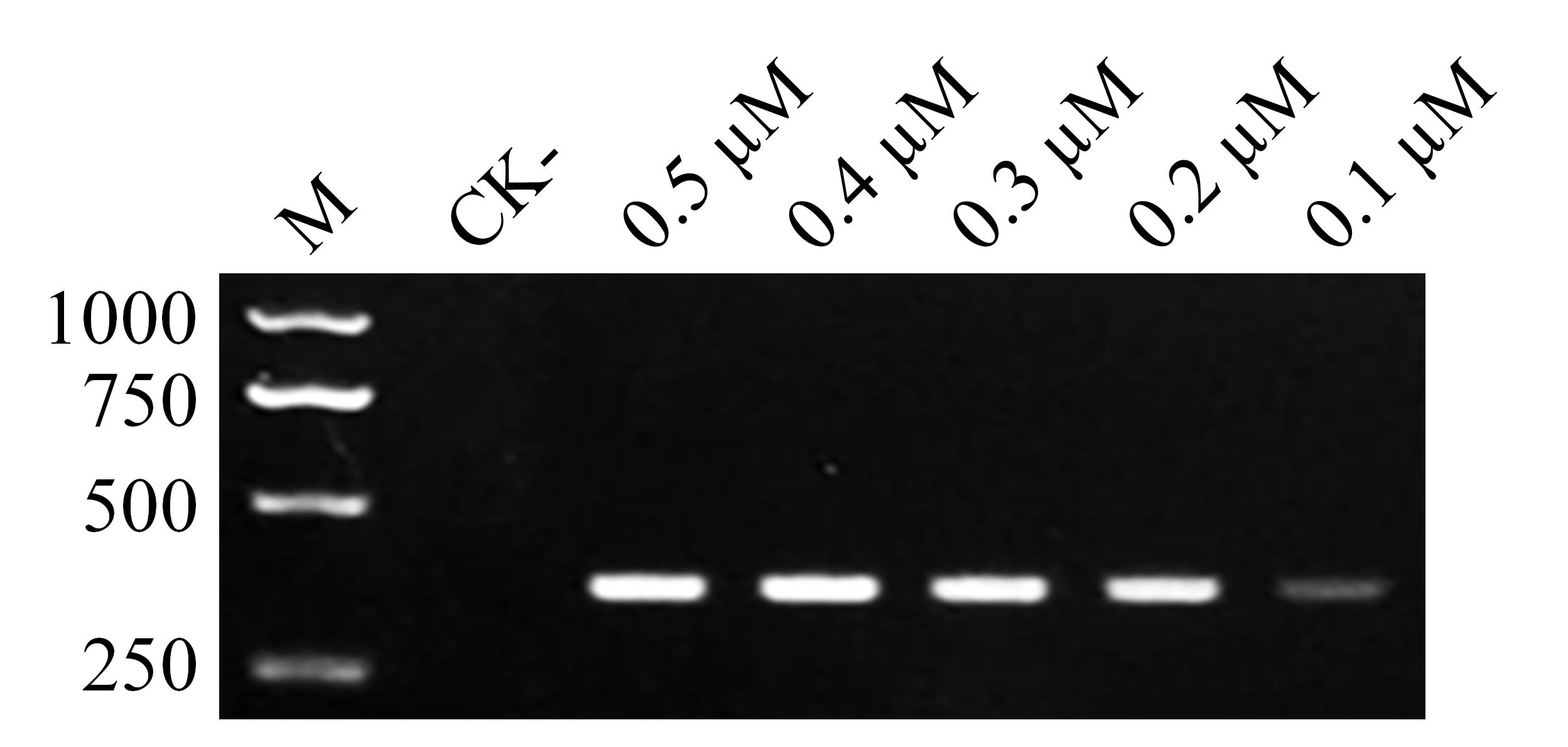

Supplement: Supplementary file 3 [file Image_3.jpeg]

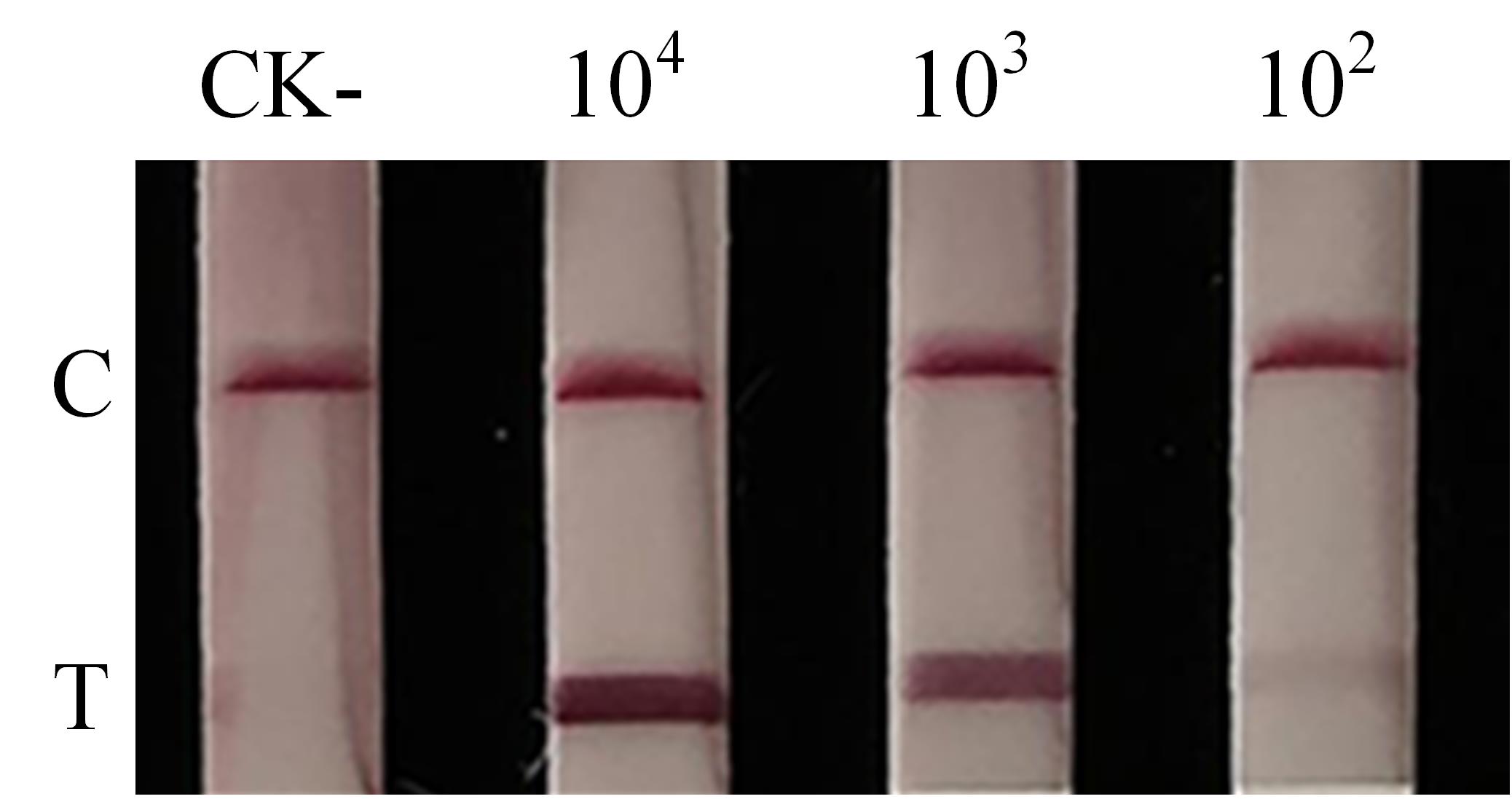

Supplement: Supplementary file 4 [file Image_4.jpeg]

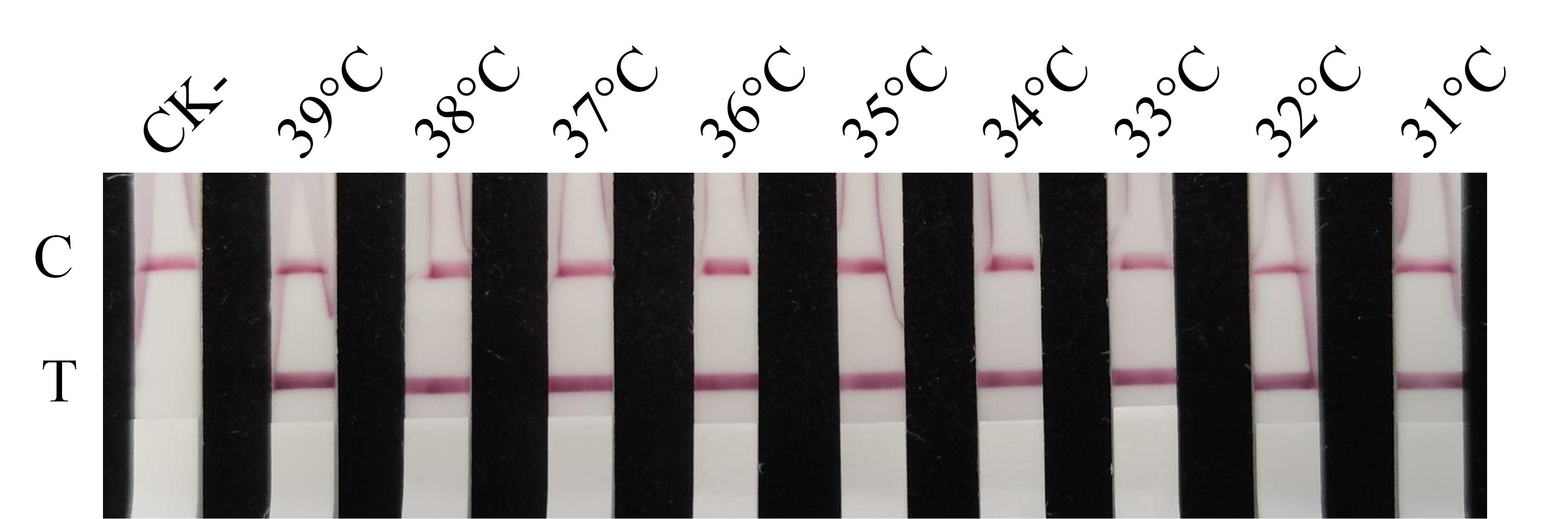

Supplement: Supplementary file 5 [file Image_5.jpeg]

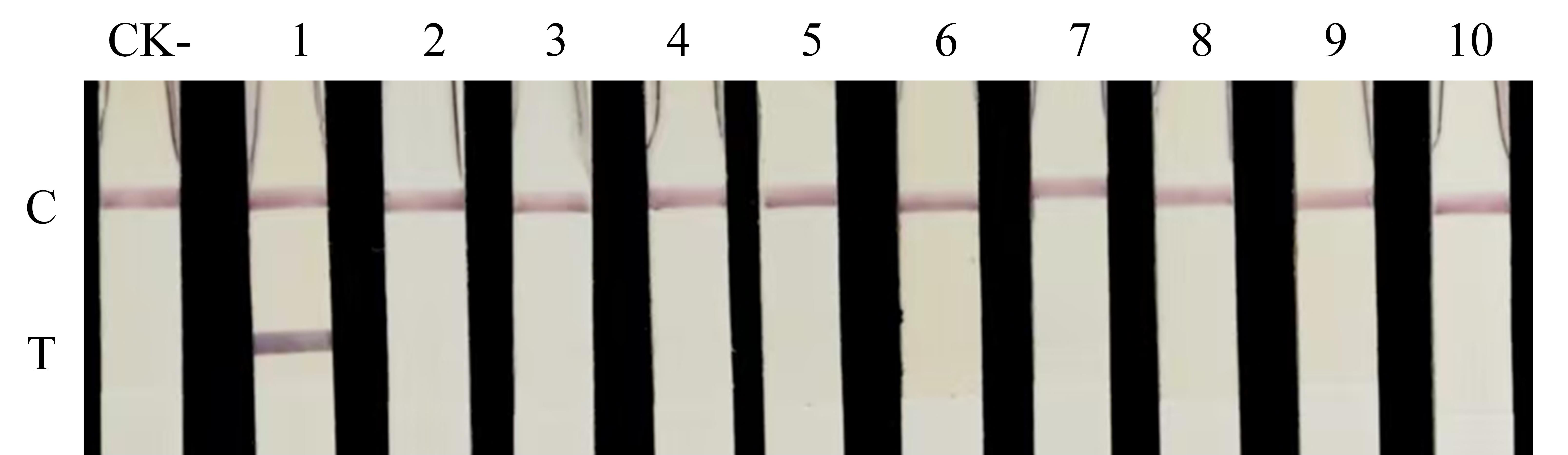

Supplement: Supplementary file 6 [file Image_6.jpeg]
